# Revisiting the interconnection between lipids and vitamin K metabolism: insights from recent research and potential therapeutic implications: a review

**DOI:** 10.1186/s12986-023-00779-4

**Published:** 2024-01-03

**Authors:** Jing Tan, Ying Li

**Affiliations:** 1 Department of Hematology, The Third People’s Hospital of Chengdu, Chengdu, Sichuan China; 2 School of Medicine, North Scihuan Medical College, Nanchong, Sichuan China

**Keywords:** Vitamin K, Lipids, Metabolism, Transport, Cholesterol

## Abstract

Vitamin K is a lipophilic vitamin, whose absorption, transportation, and distribution are influenced by lipids. The plasma vitamin K level after supplementation is predominantly a lipid-driven effect and independent of existing vitamin K status. However, previous studies examining the efficacy of vitamin K supplementation often overlooked the influence of lipid levels on vitamin K absorption, resulting in inconsistent outcomes. Recent research discovered that impaired transportation of vitamin K2 within uremic high-density lipoproteins (HDL) in individuals with uremia might elucidate the lack of beneficial effects in preventing calcification observed in multiple trials involving menaquinone-7 (MK-7) supplementation among patients with chronic kidney disease. Clinical findings have shown that drugs used to regulate hyperlipidemia interact with the vitamin K antagonist warfarin, because cholesterol and vitamin K share common transport receptors, such as Niemann-Pick C1-like 1 (NPC1L1) and ATP-binding cassette protein G5/G8 (ABCG5/ABCG8), in enterocytes and hepatocytes. Additionally, cholesterol and vitamin K share a common biosynthetic intermediate called geranylgeranyl pyrophosphate (GGPP). It is important to note that statins, which hinder cholesterol synthesis, can also impede vitamin K conversion, ultimately impacting the functionality of vitamin K-dependent proteins. Furthermore, certain studies have indicated that vitamin K supplementation holds potential in managing hyperlipidemia, potentially opening a novel avenue for controlling hyperlipidemia using dietary vitamin K supplements. Therefore, attaining a more comprehensive understanding of the intricate interplay between vitamin K and lipids will yield valuable insights concerning the utilization of vitamin K and lipid regulation.

## Introduction

Vitamin K is well known as an essential factor in blood coagulation. The letter “K” stands for “Koagulation”, a German term for coagulation [1]. Studies on various vitamin K-dependent proteins have revealed that vitamin K is involved in bone and cardiovascular physiology, inflammation, cancer prevention and endocrine function [2]. Vitamin K is not a single compound, but rather a group of similar compounds with important physiological functions. Natural vitamin K can be classified into two types: vitamin K1(phylloquinone) and vitamin K2(menaquinone) [3]. The main source of phylloquinone is plant sources, whereas menaquinone can be found in fermented foods such as soybeans, cheese, and animal sources [4]. As lipid-soluble substances, all forms of vitamin K are absorbed by the epithelial cells of the small intestine, drained into the circulation after entering the lymphatic vessels with chylomicrons (CMs), and transported to various organs and tissues by plasma lipoproteins [5]. The different lipophilicities of various types of vitamin K lead to preferential accumulation and utilization of phylloquinone in the liver and menaquinone in extra-hepatic tissues [6].

The transport capacity of lipoproteins for vitamin K may directly affect the clinical effectiveness of vitamin K supplementation. A recent study suggested that reduced transportation and uptake of MK-7 in uremic HDL in uremia may explain why many clinical trials in chronic kidney disease (CKD) patients have failed to demonstrate any significant benefit of vascular calcification with MK-7 supplementation [7].

In addition to lipid-related absorption and transportation of vitamin K, shared transporters, including scavenger receptor class B type I (SR-I), cluster-determinant 36 (CD36) [8], ABCG5/ABCG8, and NPC1L1, specifically ABCG5/ABCG8 and NPC1L1, are essential in regulating the absorption and excretion of cholesterol and vitamin K. The transporter NPC1L1 is commonly engaged in both cholesterol and vitamin K absorption. Consequently, it is conceivable that the import of vitamin K may be influenced by cholesterol via NPC1L1, as both substrates compete for the usage of this transporter [9]. A recent study identified that ATP-binding cassette protein G5/G8 (ABCG5/ABCG8) controls cholesterol and vitamin K excretion in enterocytes and hepatocytes [10]. These findings help elucidate the mechanism underlying the drug-drug interaction observed between ezetimibe (a cholesterol transport inhibitor) and warfarin (a vitamin K antagonist).

In addition to their shared transporters, cholesterol and vitamin K subtype menaquinone (MK-4) utilize the same biosynthetic intermediate. Cholesterol synthesis inhibitors, statins reduce not only cholesterol synthesis but also the prenyl-intermediate levels, thereby hindering the conversion of dietary phylloquinone into MK-4. Harshman et al. found that atorvastatin treatment in mice reduced endogenous MK-4 formation in the kidney [11]. Although statins are widely used to treat atherosclerotic cardiovascular disease, paradoxically increased vascular calcification has been verified in many clinical studies [12–14]. Statins reduce the synthesis of MK-4, which is a crucial activator of calcification suppressor matrix Gla protein (MGP), and may contribute to the statin-associated calcification.

Mounting evidence have corroborated the reciprocal relationship and influence between vitamin K and lipids, which would affect their respective functions. Studies investigating clinical effects of vitamin K, and drug-drug interaction between vitamin K and lipid-lowering drugs should take into consideration the interplay between vitamin K and lipids. This review is an analysis of the scientific literature from the PubMed, Google Scholar, and Web of Science databases on the interplay between lipids and vitamin K metabolism.

## Vitamin K subtypes and sources

Vitamin K is a lipid-soluble micronutrient that naturally occurs in two isoforms, namely vitamin K1 (phylloquinone), which is predominantly involved in the regulation of coagulation cascades, and vitamin K2 (menaquinone), which exerts beneficial effects on bone and cardiovascular health. phylloquinone has a phytyl side chain with one double bond and is found exclusively in plants, especially green leafy vegetables. It accounts for 75–90% of dietary vitamin K intake [3], with high concentrations in kale, spinach, and leeks [15]. Vitamin K2 consists of various isoforms with varying amounts of isoprenoid units connected to the menaquinone 3-position isoprene side chain. These isoforms are denoted as MK-n (MK-2, MK-4, MK-7, etc.), with ‘n’ indicating the length of the side chain. The majority of MK-n isoforms are produced by gut microbiota, which include aerobic, facultatively anaerobic, and obligate anaerobic bacteria. The main dietary sources of long-chain MK-n are natto (MK-7) [16], chicken (MK-4) [17], and cheese (MK-8, MK-9) [18]. In Western diets, vitamin K2 contributes only 10–25% of total vitamin K intake [19]. Notably, despite variances in intake of dietary sources such as phylloquinone or different menaquinones (MKs), the principal menaquinone present in mammalian tissues is MK-4 [20]. The origin of endogenous MK-4, a vitamin K2 isoform, remains controversial. An early study showed that phylloquinone, MK-4 and MK-7 undergo conversion process to MK-4 [21]. During the intestinal absorption process, the side chain of the consumed vitamin K is removed, and menadione serves as a circulating precursor for the formation of MK-4 in the specific tissues. UBIAD1, a cholesterol-synthesizing enzyme, has been identified as the underlying mechanism responsible for MK-4 accumulation in tissues by catalyzing phylloquinone conversion into MK-4 [21]. Recently, Ellis et al. found that dietary phylloquinone, MK-4, MK-7, and MK-9 serve as precursors to tissue MK-4 in mice [20]. Despite dietary variations in the type of vitamin K provided, the data indicated that tissue concentrations of MK-4 in the kidney, adipose tissue, reproductive organs, bone, and pancreas were consistent across groups that received equimolar vitamin K supplementation.

## Vitamin K absorption is regulated by lipids and shares transporters with cholesterol

Unlike fat-soluble vitamins A and D, there is no specific plasma carrier protein for vitamin K. Instead, it is mainly transported in plasma by lipoproteins [22]. Shearer and Newman [23] proposed a model for intestinal vitamin K absorption based on the principle of fat absorption. According to this model, phylloquinone and MK-7 form mixed micelles with both dietary triacylglycerols and bile salts in the intestine. These micelles then bind to chylomicrons (CMs) that contain apolipoprotein A (apoA) and apolipoprotein B-48 (apoB-48). The CMs are secreted into the lymphatic system and enter circulation through the thoracic duct. They acquire apolipoprotein C (apoC) and apolipoprotein E (apoE) from HDL, and lipoprotein lipase (LPL) hydrolyzes multiple triacylglycerols, apoA, and apoC from the CMs, resulting in the formation of chylomicron remnants (CRs) that re-enter circulation.

ATP-binding cassette transporter A1 (ABCA1) is a key protein involved in HDL formation and mediates cholesterol efflux to HDL. Studies have shown significantly lower levels of fat-soluble vitamins A, E, and K1 in ABCA1 knockout mice compared to wild-type mice [24, 25]. NPC1L1 protein is highly expressed in the brush border of small intestinal epithelial cells and is recognized as a crucial player in intestinal cholesterol absorption and a molecular target of ezetimibe [26]. Tappei et al. [9] found that NPC1L1-overexpressing human colorectal cancer cells exhibited higher uptake of phylloquinone than controls, and that NPC1L1 inhibitor ezetimibe inhibited phylloquinone uptake in a dose-dependent manner. Co-administration of ezetimibe and warfarin greatly reduces vitamin K levels, which increases the risk of bleeding by extending prothrombin time [9]. A recent study has identified the role of ATP-binding cassette protein G5 (ABCG5)/ABCG8 effluxes sterols and vitamin K in the enterocytes and hepatocytes. Thus, cholesterol and plant sterols may affect vitamin K transport by ABCG5/ABCG8, because they appear to be substrates of ABCG5/ABCG8 [10]. NPC1L1 is responsible for the absorption of both cholesterol and vitamin K, while ABCG5/ABCG8 facilitates the transport of cholesterol and vitamin K out of cells. Further investigations are warranted to investigate the effects of cholesterol-lowering drugs on vitamin K levels in the body, as well as the potential impact of vitamin K supplementation on cholesterol levels. Additionally, it would be worthwhile to explore how these interactions ultimately contribute to overall health outcomes.

## Vitamin K transportation and uptake are lipoprotein-dependent

Lipid transport involves the movement of lipid molecules between cell membranes or tissues and is primarily mediated by apolipoproteins. Apolipoproteins are crucial carriers responsible for the transport of lipids such as triglycerides (TG) and cholesterol, and are vital components of lipid metabolism and hormone metabolism. The primary carrier of phylloquinone is the triglyceride-rich lipoprotein (TRL) fraction, while smaller amounts are found in low-density lipoprotein (LDL) and high-density lipoprotein (HDL) fractions [6, 27, 28]. In Schurgers’s study [6], subjects ingested equal doses of a mixture of phylloquinone, MK-4, and MK-9. Initially, all forms of vitamin K were found to be predominantly associated with the triacylglycerol-rich lipoprotein (TGRLP) fraction. Since the TGRLP fraction is mainly cleared by the liver, most of the vitamin Kare transported to the liver. Unlike phylloquinone, both MK-4 and MK-9 were detected in TGRLP and LDL, with MK-4 additionally present in HDL. Different lipophilicities of the various vitamin K forms may contribute to differences in their plasma transport and delivery to target tissues.

In a study involving healthy adults who underwent dietary phylloquinone depletion and repletion, the plasma 2 H-phylloquinone AUC (area under the plasma concentration-time curve) exhibited a significant correlation with TG levels, irrespective of vitamin K status. The plasma response to phylloquinone intake appears to be primarily driven by lipids and is independent of the individual’s existing vitamin K status [29]. However, changes in biomarkers of vitamin K carboxylation (undercarboxylated osteocalcin and matrix gla protein) are not affected by lipids [30].

**Fig. 1:**
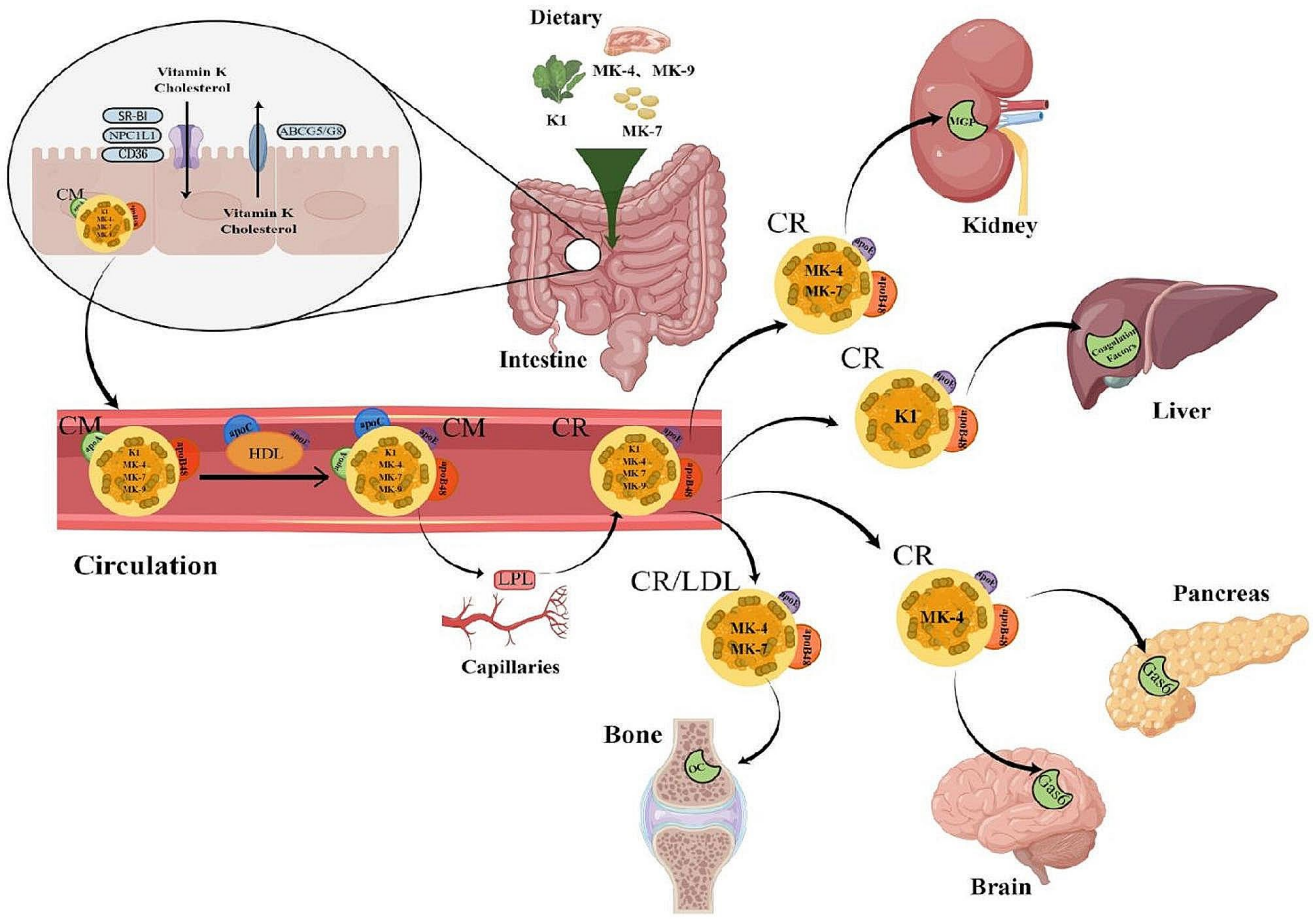
The absorption, transportation and uptake of vitamin K are lipids dependent. Green leafy vegetables are the primary dietary sources of phylloquinone. Different forms of exogenous vitamin K2, such as MK-4 found in chicken, MK-7 found in natto, and MK-9 found in chicken, are classified based on the length of the side chain. SR-BI, CD36, NPC1L1 and ABCG5/ABCG8 play a primary role in facilitating the intestinal transport of phylloquinone and cholesterol. The absorption of vitamin K and cholesterol is facilitated by SR-BI, CD36 and NPC1L1 protein, while the efflux of vitamin K and cholesterol out of cells is controlled by ABCG5/ABCG8. These mixed micelles are absorbed by intestinal epithelial cells and processed into chylomicrons that contain both apoA and apoB-48. Upon entering the blood, chylomicrons acquire additional apolipoproteins, including apoC and apoE from HDL. Lipoprotein lipase (LPL), found in the capillaries of muscle and adipose tissues, can cleave, and remove triglycerides from chylomicrons, leading to the formation of smaller remnants capable of re-entering circulation. The liver preferentially absorbs phylloquinone for the carboxylation of coagulation factors, whereas MK-4 is primarily transported to the pancreas and brain. In these organs, MK-4 may exhibit anti-inflammatory and neuroprotective effects through its binding to Gas 6 receptors. MK-7 is transported to the bone and kidneys where it regulates the functions of OC and MGP in calcium metabolism and vascular health. By Figdraw

Hepatocytes and osteoblasts participate in postprandial lipoprotein metabolism. CRs are taken up by both bone and liver, with CR uptake into bone accounting for almost 20% of liver uptake. Injection of CRs enriched with phylloquinone resulted in an increase in the degree of osteocalcin carboxylation in vivo, while total osteocalcin concentrations remained unchanged. These findings provide functional evidence supporting the utilization of vitamin K by osteoblasts through CR uptake [31]. Osteoblasts express high levels of CR receptor LRP1, which facilitates uptake of CRs and vitamin K into osteoblasts stimulated by the gamma-carboxylation of osteocalcin [32]. The absorption, transportation and uptake of vitamin K are summarized in Fig. 1.

## Vitamin K supplement potentially regulates hyperlipidemia

A study conducted on rats showed that long-term supplementation of phylloquinone or MK-4 significantly decreased the total fat accumulation, and serum triglycerides were reduced by 48% in the phylloquinone group and 29% in the MK-4 group compared with the control [33]. Nikolaos et al. recently found phylloquinone intake/body weight correlated negatively with serum triglycerides, positive association was found between phylloquinone intake/body weight ratio and HDL levels. Moreover, an increase in dietary intake of vitamin K2 was associated with a reduction in LDL-C levels [34]. In a randomized, double-blind, placebo controlled clinical trial, administration of 90 µg MK-7 daily for 8 weeks in patients with polycystic ovary syndrome (PCOS) led to significant decreases in serum TG levels, and reduced waist circumference and body fat mass along with increasing skeletal muscle [35]. Like other dietary supplement for managing chronic diseases [36, 37], there is a growing body of research on the use of vitamin K supplementation for the management of hyperlipidemia [38, 39]. Multiple intervention studies have examined the impact of vitamin K on hyperlipidemia, with some studies reporting a reduction in lipid levels while others have not observed any protective effect (Table 1.). The discrepancies could be attributed to the type of vitamin K used, duration of intervention, and the population studied. Future research should aim to identify the specific type of vitamin K that is most effective for blood lipid intervention in different populations, as well as the optimal intervention duration and population groups that may benefit from its use.

**Table 1:** Interventional studies of vitamin K on lipids profile

Intervention	Subjects	Dosage	Duration	Lipid	Findings	Ref
Phylloquinone, MK-4	Rat	600 mg/kg/d	3 months	TG	Phylloquinone: ↓TG by 48% MK-4: ↓TG by 29%	[33]
MK-7	Human	90 µg/d	8 weeks	TG	↓TG (p = 0.003)	[35]
Phylloquinone	Human	10 mg/d	8 weeks	TG, LDL-C,HDL-C, TC	No significant changes in lipid profile	[40]
Vitamin K2	Human	52 µg/d	12 weeks	TG,LDL-C,HDL-C,TC	No significant changes in lipid profile	[41]

Patients with chronic kidney disease (CKD) are at a high risk of arterial calcification or stiffness, which leads to increased cardiovascular mortality and morbidity [42, 43]. More than 80% of hemodialysis patients have vitamin K deficiency, which is closely related to vascular calcification [44]. Moreover, the supplement of vitamin K reduced the undercarboxylated MGP [45]. However, several randomized controlled trials with high-dose MK-7 supplements in patients with CKD have failed to show cardiovascular benefits [46–48].

The paradoxical phenomenon of the lack of cardiovascular benefit from MK-7 supplementation in CKD patients may be explained by altered vitamin K biodistribution and metabolism related to lipids in both experimental models and CKD patients, as demonstrated in a recent study [7]. This study found that the uptake of phylloquinone was reduced in all lipoprotein fractions, and MK-7 uptake in HDL was significantly lower in the hemodialysis group compared to healthy controls. To further investigate this, HDL particles were isolated and incubated in vitro using phylloquinone, MK-4, and MK-7. The results showed that the uptake of all three forms of vitamin K was significantly increased in the HDL fraction of the healthy control group, while the HDL fraction in the hemodialysis group exhibited almost no uptake of MK-7. This reduced transportation and uptake of MK-7 in uremic HDL may explain the failure of several trials to demonstrate a significant benefit of vascular calcification with vitamin K2 supplementation in patients with CKD. Future studies could explore whether higher doses can compensate for the altered vitamin K metabolism and transport observed in CKD patients.

## The dual effect of statins on both cholesterol and vitamin K contributes to statin-induced calcification

**Fig. 2:**
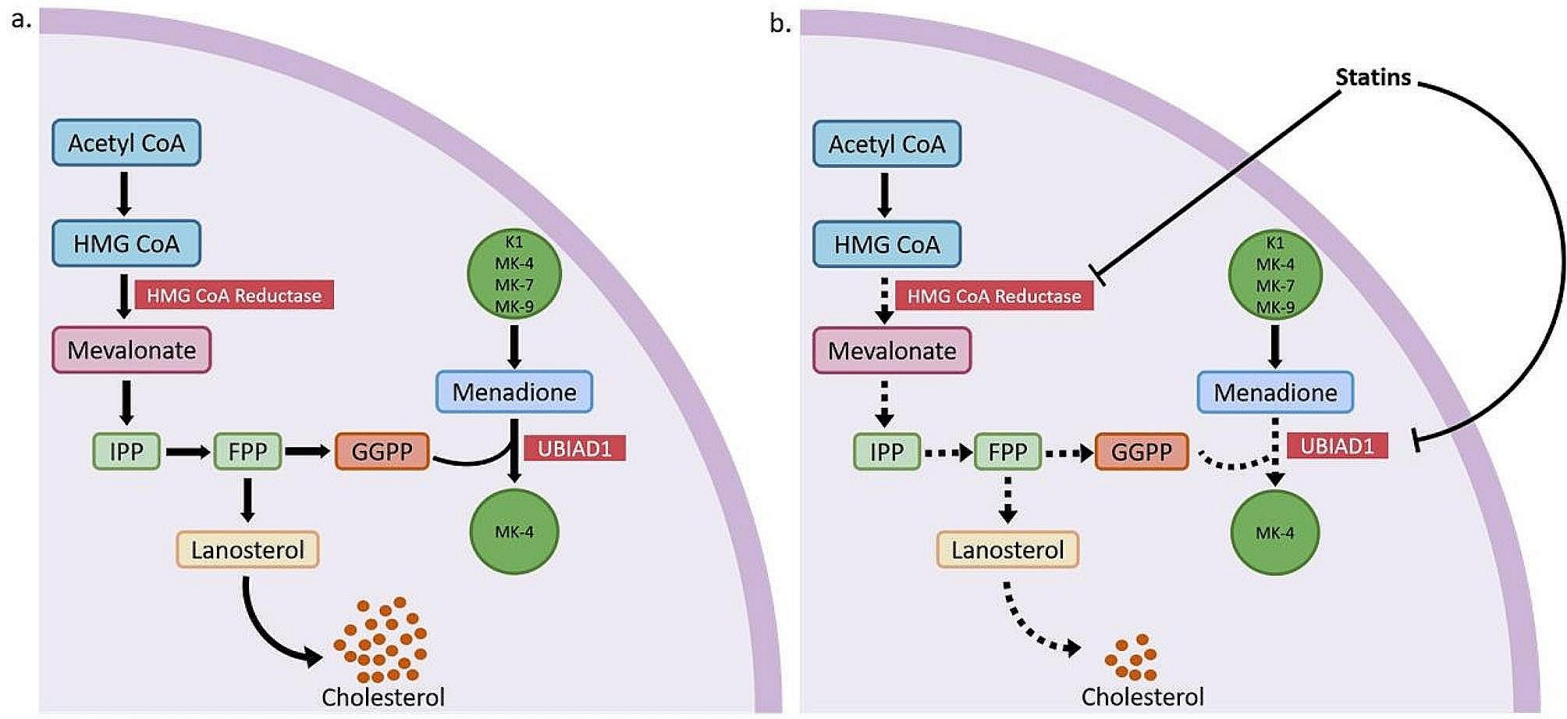
Statins inhibit synthesis of cholesterol and MK-4. **(a)** The synthesis of cholesterol begins with the conversion of acetyl-CoA, a substance formed through the breakdown of glucose or fatty acids within the mitochondria, into 3-hydroxy-3-methylglutaryl-CoA (HMG-CoA) in the cytoplasm. This conversion is catalyzed by the enzyme HMG-CoA synthase. HMG-CoA reductase then converts HMG-CoA into mevalonate, which is further modified into farnesyl pyrophosphate (FPP). FPP undergoes cyclization steps to synthesize squalene, which is later converted into lanosterol. FPP can also be transformed into GGPP, a precursor for MK-4 synthesis. UBIAD1 plays a major role in this process as it facilitates the conversion of GGPP into MK-4. **(b)** By inhibiting the HMG-CoA reductase enzyme, statins reduce the levels of mevalonic acid, a precursor for cholesterol and isoprenoids, including GGPP. Since UBIAD1 uses GGPP as a substrate for vitamin K2 (MK-4) biosynthesis, the use of statins may indirectly decrease GGPP production, thereby affecting the availability of MK-4. However, despite the excessive abundance of GGPP, the levels of generated MK-4 were not fully restored, suggesting the likely direct inhibition of UBIAD1-mediated MK-4 synthetase activity by statins

Statins exert their lipid-lowering effects by inhibiting 3-hydroxy-3-methyl glutaric acid monoacyl coenzyme A (HMG-CoA) reductase, a key enzyme in cholesterol synthesis. Studies suggest a potential link between high-dose statin therapy and increased vascular calcification, especially in patients with advanced CKD or those undergoing dialysis [12, 49]. One possible explanation is that statins may interfere with the synthesis of vitamin K-dependent proteins, which play a role in preventing calcification. Statins inhibit the activity of HMG-CoA reductase enzyme, which in turn reduces the availability of mevalonic acid, a precursor for cholesterol and isoprenoids such as geranylgeranyl pyrophosphate (GGPP). GGPP is the substrate of UBIAD1, an enzyme that is involved in the biosynthesis of vitamin K2 (MK-4) [50] (Fig. 2a). Harshman et al. found that atorvastatin significantly reduced the levels of total MK-4 in the kidneys of mice by approximately 45% compared to untreated mice [11]. The synthesis of MK-4 in UBIAD1 was found to be intricately linked to intercellular cholesterol synthesis [51]. Cholesterol biosynthesis occurs via the mevalonate pathway, wherein geranyl pyrophosphate (GPP), farnesyl pyrophosphate (FPP), and squalene serve as intermediates. Of these, MK-4 is simultaneously synthesized by UBIAD1 with GGPP, which is derived from FPP via the action of GGPP synthetase. Therefore, statins may indirectly decrease the synthesis of GGPP, which can affect the availability of MK-4. However, atorvastatin reduced endogenous MK-4 formation in the kidney, but not other organs, and further research is needed to understand potential regulatory mechanisms and the unique functions of MK-4 in the kidney [11]. Additionally, statins have been shown to directly inhibit the MK-4 synthetase UBIAD1, which is responsible for the production of MK-4 [52] (Fig. 2b). In summary, statins inhibit HMG-CoA reductase, leading to a decrease in the availability of intermediates required for the biosynthesis of MK-4. Additionally, statins may also hamper the activity of UBIAD1, thus preventing it from producing MK-4. Insufficient vitamin K production will lead to insufficient carboxylation of vitamin K-dependent protein MGP, which cannot effectively inhibit vascular calcification. Particularly in the setting of multiple pro-calcification factors, the endogenous vitamin K production deficiency caused by statins cannot effectively inhibit calcification formation, which is an important reason why statins promote vascular calcification. Future studies may explore the effect of vitamin K supplementation on vascular calcification in patients treated with statins.

## Conclusions

As a fat-soluble vitamin, the transport and absorption of vitamin K are highly dependent on lipids. Vitamin K shares common cell surface transporters with cholesterol, so interventions aimed at the cholesterol absorption pathway may also interfere with vitamin K levels. Conversely, supplementing vitamin K can also influence lipid levels, as evidenced by the regulation of hyperlipidemia with vitamin K supplementation in PCOS patients, which offers a potentially safe dietary solution for treatment of this condition. Although numerous pathological conditions, including osteoporosis, vascular calcification, are characterized by a low level of vitamin K, which have led to a growing interest in clinical research exploring the possibility of alleviating or preventing these chronic diseases through vitamin K supplementation. However, the results of recent clinical studies on vitamin K supplementation have been inconsistent. One important reason for this is that early animal experiments have revealed that plasma vitamin K response to vitamin K supplementation is a lipid-driven effect and independent of existing vitamin K status. It was not until recent research that the impaired utilization of MK-7 by HDL in uremia patients was discovered in clinical setting. The decreased transportation and absorption of MK-7 in uremic HDL, observed in uremic patients, may elucidate the reasons behind the inconclusive results of numerous clinical trials involving the administration of vitamin K2 supplementation in CKD patients to mitigate vascular calcification. The strength of our work is that we propose a putative mechanism explaining the impact of statins on vascular calcification: by decreasing the production of MK-4, which activates the inhibitor of vascular calcification MGP. Further investigations are needed to validate this hypothesis.

Overall, understanding the close relationship between vitamin K and lipids will provide better direction for studying and utilizing vitamin K, and may also offer a new intervention for hyperlipidemia.

## Data Availability

Not applicable.

## Abbreviations

CMs: chylomicrons
NCP1L1: Niemann-Pick C1-like 1
UBIAD1: UbiA prenyltransferase containing 1
GGPP: geranylgeranyl pyrophosphate
CKD: chronic kidney disease patients
CRs: chylomicron remnants
TRL: triglyceride-rich lipoprotein
TGRLP: triacylglycerol-rich lipoprotein
AUC: area under the plasma concentration-time curve
PCOS: polycystic ovary syndrome
GPP: geranyl pyrophosphate
FPP: farnesyl pyrophosphate
